# *Syzygium cumini* Fruit Extract and Quercetin Ameliorate Cadmium-Induced Ovarian Apoptosis in Rats Via miRNA- 204 - 5p-Mediated Bcl- 2 Upregulation and Bax/Caspase 9/Caspase 3 Downregulation

**DOI:** 10.1007/s12011-025-04632-y

**Published:** 2025-05-14

**Authors:** Walaa I. E. Abdel-Badeea, Ahmed Abdel-Wahab, Ahmed A. Mahmoud, Abdel-Razik H. Abdel-Razik, Eman Z. Attia, Usama R. Abdelmohsen, Kamel M. A. Hassanin

**Affiliations:** 1 Biochemistry Department, Faculty of Veterinary Medicine, Minia University, 61519 Minia, Egypt; 2 Physiology Department, Faculty of Veterinary Medicine, Minia University, 61519 Minia, Egypt; 3 Chemistry Department, Faculty of Sciences, Minia University, 61519 Minia, Egypt; 4 Histology Department, Faculty of Veterinary Medicine, Beni-Suef University, Beni-Suef, 62511 Egypt; 5 Pharmacognosy Department, Faculty of Pharmacy, Minia University, 61519 Minia, Egypt; 6 Pharmacognosy Department, Faculty of Pharmacy, National Minia University, 61111 New Minia, Egypt; 7 Pharmacognosy Department, Faculty of Pharmacy, Deraya University, 61111 New Minia, Egypt

**Keywords:** *Syzygium*, Quercetin, Cadmium, Apoptosis, MicroRNAs

## Abstract

The correlation between exposure to environmental pollutants and adverse impacts on fertility has been documented. Cadmium (Cd) is one of the most toxic elements that impairs female reproductive capacity. *Syzygium cumini* is a plant that contains polyphenols with strong antioxidant properties. Our study aimed to investigate the possible protective effects of *Syzygium cumini* fruit extract (SCFE) and quercetin (QU) against Cd-induced ovarian toxicity and apoptosis in rats assisted by measuring oxidative stress markers, hormonal profile, intrinsic apoptotic pathway, miRNA-204-5p, and histopathology. For that, 45 adult female rats were divided randomly into five groups; control, cadmium chloride (CdCl_2_), CdCl_2_ + QU, CdCl_2_ + SCFE200, and CdCl_2_ + SCFE400, and the experiment lasted 35 days. Cadmium exposure induced irregular estrus cycle, and oxidative challenges, as evidenced by increased malondialdehyde and decreased antioxidant indicators that include superoxide dismutase, catalase and reduced glutathione, and hormonal imbalances, including reduced follicle-stimulating hormone, luteinizing hormone, anti-mullarian hormone, estrogen, and progesterone levels. Additionally, Cd triggered ovarian apoptosis through upregulating *miRNA- 204 - 5p* expression that in turn downregulated *Bcl- 2* and upregulated the members of intrinsic mitochondrial apoptotic pathway including *Bax* and *caspases 3*, *9*. The histopathological findings, morphometric and lesion scoring in ovarian, and uterine tissues confirmed the negative impacts of Cd. Interestingly, treatment with QU or SCFE, especially at the higher dose (400 mg/kg), significantly ameliorated these Cd-induced adverse effects, suggesting their potential as protective agents against ovarian toxicity.

**Supplementary Information:**

The online version contains supplementary material available at 10.1007/s12011-025-04632-y.

## Introduction

One of the biggest issues facing contemporary civilization is infertility, which affects a large number of people worldwide. Infertility has been associated with a number of chemicals, including heavy metals [1]. One of the major environmental contaminants is cadmium (Cd), which ranks seventh on the Agency for Toxic Substances and Disease Registry's (ATSDR) substance priority list for substances that are hazardous to living things [2].

Cadmium is commonly utilized in industry, particularly in the chemical, electronics, and nuclear sectors [3, 4]. Water and nutritional items such as cereals, fruits, vegetables, leafy vegetables, and shellfish all contain Cd, which enters the body via the lungs and digestive tract [5], making Cd exposure a major public health concern. Additionally, Cd is not biodegradable; therefore, it can enter the food chain and accumulate in the body after being absorbed. This can result in irreparable damage to a variety of organ systems, particularly the reproductive ones [6, 7]. Infertility is largely caused by decreased germ cell production [8, 9], which was observed to be triggered by Cd toxicity [10]. It has been shown that Cd has an impact on female’s reproductive system which is manifested by subfertility, infertility, and growth retardation in the intrauterine region [11].

Cd exposure can lead to oxidative stress because it generates more free radicals than antioxidants can neutralize. Consequently, this will cause levels of reactive oxygen species (ROS) to rise [12, 13]. Exposure to ROS may cause reproductive damage, including lipid peroxidation, DNA damage, and apoptosis [14, 15] Within the female reproductive system, ROS damages oocytes and prevents ovarian growth, which results in subfertility [16]. Elevations in reactive oxygen species (ROS) levels are usually accompanied by an abundance of malondialdehyde (MDA), while levels of antioxidant markers such as reduced glutathione (GSH), catalase (CAT), total antioxidant capacity (TAC), and superoxide dismutase (SOD) are depressed [17]. These findings proved that Cd could cause oxidative stress, which shortens the reproductive lifespan causing infertility, as reported by the study of Aitken [18].

It has been demonstrated that Cd induces apoptosis of oocyte granulosa cells (OGC) by chromatin condensation and DNA breakage [19], which in turn suppresses the expression of B-cell lymphoma 2 (Bcl- 2) gene. The Bcl- 2 family proteins are involved in the intrinsic apoptotic process. This family can be further divided into three classes based on the intracellular structures and functions they perform. From these classes, Bcl- 2 antagonist/killer (BAK) and Bcl- 2-associated X protein (Bax) are proapoptotic Bcl- 2 family members, but the Bcl- 2 gene is an antiapoptotic one [20].

The expression of Bcl- 2 gene was found to be under the control of a group of microRNAs (miRNAs) [21]. miRNAs, the most common form of noncoding RNA, are critical for numerous physiological and cellular processes, which have attracted a lot of attention, particularly with ovarian functions via OGC [22]. Hundreds of miRNAs have been implicated in Cd toxicity, which has been shown to cause harm to many organs, reproductive toxicity, malignant transformation, and aberrant DNA damage [23]. As a result, we chose to study the putative role of miRNAs in ovarian apoptosis mediated by Cd.

Naturally occurring flavonoid quercetin (QU) is present in a variety of fruits, vegetables, and plant-based diets. It has gained recognition for its many health benefits, which include anti-inflammatory, antioxidant, and potentially anticancer impacts [24]. Quercetin is a bioactive molecule with significant antioxidant properties that benefit the human body and a variety of ailments [25]. By upregulating the relative protein expression of certain genes of oxidative markers (SOD- 1 and CAT), QU enhanced the ovary’s antioxidant capacity [26].

Recently, some plants containing polyphenolic chemicals like *Moringa oleifera* leaf extracts [27], chokeberry, black and green tea, blueberry, and rosemary [28] have demonstrated their efficacy as natural antioxidants and demonstrated a notable degree of protection against the toxicity of Cd [29, 30]. Additionally, locust bean gum-stabilized nanogold functionalized with *Phyllanthus reticulatus* anthocyanins (LBG/PRA-NG) demonstrated anticancer activity attributed to the anthocyanins and gum serving as a bio-reductant and stabilizer, respectively, exhibiting exceptional antioxidative properties with IC50 values of 35.44 μg/mL against DPPH and 24.55 μg/mL against ABTS [31]. Furthermore, the lipophilic extract of *C. odorata* aerial parts (COLE) indicated significant and selective anticancer efficacy against human breast and colon cancer cells, along with enhanced antibacterial and antioxidant attributes [32]. The lipophilic portion of *E. camaldulensis* leaf extract contains pharmacologically significant active compounds with considerable potential for addressing illness states associated with oxidative stress, protein aggregation, and MCF- 7 breast cancer [33].

The most prevalent of these kinds of plants is *Syzygium cumini* which is a tropical/subtropical tree having a variety of therapeutic uses, most notably antidiabetic, anticancer, and antibacterial effects. It is also referred to as black plum, jamun, jambolana, Indian blackberry, Java plum, or Malabar plum [34]. Additionally, it is a great source of several bioactive substances that successfully resist oxidative damage, including tannins, stilbenes, and phenolic metabolite [35, 36]. According to previous studies, the bright purple color of jamun is caused by anthocyanins, delphinidin, petunidin, and malvidin-diglucosides found in its pulp [37]; glucose, fructose, citric acid, mallic acid, gallic acid, delphinidin- 3-gentiobioside, malvidin- 3-laminaribioside, petunidin- 3-gentiobioside, and cyanidin diglycoside are all abundant in fruits [38]. Jamun pulp is nutrient-dense and contains free amino acids like alanine, asparagine, tyrosine, glutamine, and cysteine; carbohydrates like glucose, mannose, sucrose, maltose, fructose, and galactose; minerals like sodium, potassium, calcium, phosphorous, iron, and zinc; water-soluble vitamins like ascorbic acid, thiamine, and niacin; and citronellol, geraniol, herol oxide, hotrienol, linalool, linalool oxide, nerol, and β-phenylethanol [39, 40].

This study selected *Syzygium cumini* fruit extract based on findings by Ahmed et al. [41], which indicated that the fruit pulp (edible portion, including peel) and seeds possess significant sources of natural bioactive compounds with remarkable antioxidant properties particularly total phenolics, flavonoids, and tannins. The findings of that study were pioneering to move to advanced research on the utilization of *S. cumini* as an alternative to artificial antioxidants in food products. The research conducted by Abdel Aziz et al. [42] examined the effects of small (10 mg/kg) and large (50 mg/kg) dosages of QU against atrazine-induced testicular toxicity. The study revealed that the smaller dosage served as a potent antioxidant, safeguarding the testes from atrazine toxicity, but the larger dosage functions as a pro-oxidant. In this study, we decided to use a low dose of 10 mg/kg to examine its potential protective effects against cadmium-induced ovarian toxicity.

Thus, the purpose of this study was to assess the safeguarding effects of QU and *Syzygium cumini* fruit extract (SCFE) against reproductive harm caused by Cd in adult female rats. To accomplish this, we assessed markers of oxidative stress in ovarian tissues, specifically focusing on the intrinsic ovarian apoptotic pathway including miRNA- 204 - 5p, Bcl- 2/Bax/caspases 3,9 pathway. Additionally, we performed histological analyses of both the ovaries and uterus.

## Material and Methods

### Chemicals

Cadmium chloride 99.99% based on trace metals analysis was purchased from Sigma-Aldrich, USA, and dissolved in distilled water and administered by gavage. Quercetin was purchased also from Sigma-Aldrich Company, USA, and purity of ≥ 95% (HPLC). It is water-suspended, and the suspension was prepared by suspending it in sterile distilled water to be administered by gavage. A stock from both cadmium chloride and QU was prepared every week.

### Preparation of *Syzygium cumini* Fruit Extract

Fresh *Syzygium cumini* (L.) fruits were gathered from a private garden located in El-Qeis village, Beni-Mazar city, El-Minia, Egypt, during August 2023. The authenticity of the fruits was verified by Prof. Dr. Nasser Barakat, Department of Botany, Faculty of Sciences, Minia University, Egypt. A voucher specimen (Sc- 2) has been deposited in the Herbarium of Department of Botany, Faculty of Sciences, Minia University. The fruits were properly cleaned in running tap water to remove contaminants from their surfaces, and the seeds were hand-separated from the pulp. The preparation of the SCFE was done according to the method described by Ahmed et al. [41]. Extraction of the bioactive ingredients of the fresh fruit pulps was carried out with an ethanol–water solution (2:1, V/V) as a solvent. The fresh fruit pulps (980 g) were suspended in a 4-L solvent and shaken continuously at room temperature for 5 days with an orbital shaker (SKR- 202, OMEGA, Thailand) at 50 rpm. After filtering via Whatman No. 1 filter paper in a Buchner funnel to remove the residues, the extraction was concentrated using a vacuum rotary evaporator (R- 111, Buchi, Switzerland).

For the biological investigations and LC–MS analysis, the 159-g *Syzygium cumini* residue that was recovered was divided into vials, each holding 1 g, and kept at − 20 °C. Before being delivered to animals, residue from each vial was first suspended in a normal saline solution containing 0.9% sodium chloride in order to achieve a final concentration of 1 g/mL. The leftover residue from each daily dosage was stored at 4 °C so it could be used the following day.

### The Assay of *Syzygium cumini* Fruit Extract’s In Vitro Antioxidant Activity and Phytochemical Analysis

Following a phytochemical examination of the fruit extract from *Syzygium cumini*, the following assay was used to measure the antioxidant activity in vitro:

1. Total flavonoid content was estimated using a modified colorimetric method, as previously described [43], while the total phenol content of SCFE was analyzed using the previously established method [44].
2. In order to evaluate the antioxidant activity of the extract, two methods were used:
3. The phosphomolybdate complex method [45] was employed to measure the overall reducing capabilities, with ascorbic acid serving as the reference standard.
4. As was previously disclosed [46], the scavenging potentials for free radicals of the extract were determined using spectrophotometry and the 1, 1-diphenyl- 2-picryl-hydrazyl (DPPH) method.

### LC–MS Analysis

A 3000 HPLC system (DionexUltiMate) coupled with a Thermo Scientific Exact mass analyzer (Kierlau, Germany) was used to perform high-resolution LC–ESI–MS of *Syzygium cumini* fruit extracts. The mobile phase, which was eluted at a flow rate of 300 µL/min, consisted of acetonitrile (A) and water (B) with 0.1% formic acid. Gradient elution was initiated with 10% acetonitrile (10 mL acetonitrile and 90 mL water) for 5 min, then over the course of 30 min, it was increased to 100% acetonitrile, which was held for an additional 5 min before falling back to 10% acetonitrile in the 6 th min.

The MS dataset underwent processing and data extraction using MZmine 2.20, according to the predefined settings. Then, we performed chromatogram deconvolution followed by peaks deisotoping. Normalization of the retention time was applied for chromatographic alignment and gap-filling [47]. A detailed description of the phytochemical analysis of the plant was provided in the supplementary data.

### Animals and Experimental Design

All procedures utilized on the rats in this investigation were compliant with the guidelines set out by the local Institutional Animal Care and Use Committee of Research (Approval number: IRB-FVM-MU- 2023–107).

A total of 55 adult Wistar albino female rats were used in this study. They weighed an average of 142 ± 2.30 upon arrival from the Minia University, Faculty of Medicine’s lab animal center. They were allowed 2 weeks to acclimatize before the study began. Every single one of the rats was provided with the standard rat diet, which included yellow maize “2490.00 kcal/kg,” crude fiber, fat, vitamins, minerals, and protein (68%, 3%, 7%, 1%, and 21%, respectively). This diet was given without any restrictions to the rats while they were being housed in plastic cages.

The estrous cycle was observed by daily early-morning vaginal smear cytological examination after the 2 weeks of acclimation as previously described [48]. The study only included females which had two consecutive regular cycles; irregular cyclers were not included.

The dose of SCFE used in the current study has been recognized in various traditional medicine systems and used in the treatment of various diseases and ailments in humans. Some studies used small doses such as 100 and 200 mg/kg BW [49, 50], while others used large doses such as 250, 500, and 750 mg/kg BW [51] and 400 [52]. As a result, we intended to employ two doses in this study: a small dose of 200 mg/kg and a large dose of 400 mg/kg BW to compare their protective effects.

Concerning QU, ABDEL AZIZ et al. [42] investigated small and large dosages of QU and discovered that the small one (10 mg/kg BW) is an antioxidant whereas the large one (50 mg/kg BW) acts as a prooxidant. Thus, in this investigation, we selected a low dosage of 10 mg/kg BW. Concerning CdCl_2_, we utilized a dose (5 mg/kg BW) comparable to the relevant exposed ambient level observed in the publications, including our previous work [53] and other report [54].

After identifying rats that exhibited regularity in their estrus cycle, they were randomly assigned to five groups (*n* = 9). Each group of animals received the proper treatment on a daily basis for a duration of 35 days. The period is 35 days to cover a sufficient number of estrus cycles (7 cycles), as the estrus cycle in rats is around 5 days [55], to detect the effect of the examined treatments on CdCl_2_-induced ovarian toxicity.

The groups comprised the following: (1) the control group consisted of rats that were given distilled water (DW). (2) The CdCl_2_ group consisted of rats that were given 5 mg of CdCl_2_ per kg BW dissolved in DW, as reported before [53]. (3) CdCl_2_ + QU group: according to the studies conducted by Abdel Aziz et al. [42], rats were orally administered 10 mg QU/kg BW dissolved in DW 1 h before receiving the same quantity of CdCl_2_ (5 mg per kg BW). (4) CdCl_2_ + small dose of SCFE “SCFE200”: rats were orally administered 200 mg SCFE/kg BW suspended in DW [52], 1 h before administration the same dose of CdCl_2_. (5) CdCl_2_ + large dose of SCFE group “SCFE400”: rats were orally administered a high dose of the SCFE group SCFE400, consisting of 400 mg of SCFE per kg BW, suspended in distilled water [52]. This also occurred 1 h before administering the same dosage of CdCl_2_.

### Evaluation of Estrus Cycle Regularity

To evaluate the effects of treatments on estrus cycle regularity, the animals were subjected daily to vaginal smear checkups between 08:00 and 09:00 a.m. for 2 successive cycles before the end of the experiment as previously outlined [48, 56]. Essentially, the assessment of the stage of the estrus cycle in the vaginal smear relies on identifying which of these cells (leucocytes, cornified epithelial cells, and nucleated epithelial cells) is the most abundant. Rats were considered to be in proestrus when nucleated cells dominated the cell types. The rats were found to be in the estrus stage when the predominant cell type was cornified cells; in the metestrus stage when the predominant cell type was both leukocytes and cornified cells; and in the diestrus stage when the predominant cell type was leukocytes. The regularity of estrous cycle was dichotomized into regular or irregular (regular estrous cycles: 24–48 hs of estrus,12–24-hs metestrus, 48–72 hs of diestrus, and 12–24 hs proestrus; irregular estrous cycles: 3–4-days estrus, 2–3-days metestrus, 4–5-days diestrus, and 2–3-days proestrus).

Subsequently, during the estrus period, rats were subjected to blood collection and subsequently euthanized to obtain tissue samples.

### Body Weights, Ovarian Weights, and Gonadosomatic Index

On the 35 th day (the last day of the experiment), each rat’s body weight was measured separately with a digital balance. Furthermore, following the 35 th day, animals were euthanized during the estrus period and dissection of the abdomen was performed. Then, the ovaries of each rat were removed and weighed. The gonado-somatic index (GSI) was calculated by dividing the average ovarian weights in grams by the corresponding body weights in grams and multiplying by 100.

### Serum and Tissue Samples

Upon completion of the experiment, female rats in the estrus phase were subjected to anesthesia using ketamine HCl 90 mg/kg. BW and xylazine HCl 5 mg/kg. BW according to [57]. Then, the samples of blood were collected from each rat individually, from the retro-orbital venous plexus, in a sterile and dry tubes. They were left at a room temperature for 30 min to clot and centrifuged to obtain serum samples. The sera were stored at − 20 °C till the moment of evaluating the researched parameters.

After that, the animals were euthanized by decapitation, and their abdominal cavity was incised to retrieve the necessary tissues for subsequent analyses. Each rat had its ovaries removed, and one of the ovaries was divided into two parts, one of which was used to measure the parameters related to oxidants and antioxidants. To do this, we first homogenized the ovarian portion using a tissue homogenizer in 5 mL of phosphate-buffered saline (PBS). Subsequently, we collected the supernatants and preserved them at a temperature of − 20 °C until the assessment of oxidant/antioxidant indicators. The remaining ovarian fraction was kept in an Eppendorf tube containing 0.5-mL PBS and 0.5-mL RNA*later*™ Stabilization Solution (Catalogue No.:AM7020, Thermo Fisher Scientific, MA, USA) at − 20 °C till its use for RT-qPCR analysis of the genes under investigation. The other ovary and uterine tissues were placed in neutral buffered formalin (10%) for histopathological examination.

### Hormonal Assay

By using specific ELISA kit, the hormones including follicle-stimulating hormone (FSH) (Rat FSH ELISA Kit, Catalogue No.: ER0960, Wuhan Fine Biotech Co., Ltd., Wuhan, Hubei, China), luteinizing hormone (LH) (Rat LH ELISA Kit, Catalog Number. CSB-E12654r, CUSABIO Company, USA), estrogen (Rat Estrogen ELISA Kit, Cat. No E0176Ra, Bioassay Technology Laboratory Co., Shanghai, China), progesterone (Rat Progesterone ELISA Kit; Catalogue No.: ER1255; Wuhan Fine Biotech Co., Ltd., Wuhan, Hubei, China), and anti-Mullerian hormone (AMH) (Rat AMH ELISA Kit, Catalog No: E-EL-R0640, Elabscience, USA) were all tested following the manufacturer’s instructions.

### Determination of Oxidative Stress Markers

Using particular colorimetric assay kits (Biodignostic Company, Giza, Egypt; Catalogue numbers: MD 25 29, TA 25 13, SD 25 21, GR 25 11 and CA 25 17 for MDA, TAC, SOD, GSH and CAT, respectively) and following the manufacturer’s instructions, all investigated oxidative stress markers were measured in ovarian tissue homogenates.

The MDA concentration is determined by thiobarbituric acid and MDA reacting for 30 min at 95 °C in an acidic solution. The result of this reaction is reactive thiobarbituric acid. The absorbance of the resulted colored compound was quantified at a wavelength of 534 nm.

The total antioxidant capacity (TAC) is evaluated by the reaction of antioxidants in the sample with a specific amount of externally added hydrogen peroxide (H_2_O_2_). A portion of the supplied H_2_O_2_ is removed by the sample’s antioxidants. Through an enzymatic reaction involving the conversion of 3,5,dichloro- 2-hydroxy benzenesulfonate to a colorful product (measured at 505 nm wavelength), the residual H_2_O_2_ is detected colorimetrically.

The fundamental idea of the SOD assay is based on the enzyme’s capacity to prevent the nitro blue tetrazolium dye from being reduced by phenazinemethosulfate. GSH test based on the ability of GSH to reduce 5, 50-dithiobis- 2-nitrobenzoic acid, which ultimately results in the development of a substance that is yellow. The amount of GSH is directly correlated to the absorbance that is measured at 405 nm.

To measure CAT, it initially undergoes a reaction with a certain amount of H_2_O_2_, followed by the addition of a catalase inhibitor to halt the reaction after 1 min. When horseradish peroxidase (HRP) is present, the remaining H_2_O_2_ reacts with 3, 5-dichloro- 2-hydroxybenzene sulfonic acid (DHBS) and 4-aminophenazone (AAP) to generate a pigmented compound that is inversely proportional to the amount of CAT in the original ovarian homogenate sample.

### Determination of mRNA Relative Expression Levels of Rats’ Ovarian Apoptotic, Antiapoptotic, and miRNA 204 - 5p Genes by RT-qPCR

#### Bcl- 2, Bax, Caspase 9, and Caspase 3

Following the manufacturer’s instructions, the ovarian RNA was extracted using TRI REAGENT®-RNA/DNA/PROTEIN ISOLATION REAGENT (Molecular Research Center, Inc. Cincinnati, OH., USA, Catalog number: TR 118). We utilized a T80 UV/VIS Spectrometer (PG Instruments Ltd, Beijing, China) to quantify the quantity and assess the quality of RNA. A measure of the absorbance at 260 and 280 nm was used to quantify the purity of DNA and RNA. For DNA, a ratio of 1.8 is typically considered “pure,” whereas RNA is considered pure at a ratio of 2.0. In addition, following the manufacturer’s instructions, the GoTaq® 1-Step RT-qPCR System kit (Promega Corporation, 2800 Woods Hollow Road, Madison, WI 53711 USA; Catalog number: A6020) was used to perform a one-step qPCR assay on the rats’ ovarian mRNAs of Bcl- 2, Bax, caspase 3, caspase 9, and β-actin. It includes two major steps: the first step is the synthesis of cDNA complementary to rats’ ovarian mRNAs of Bcl- 2, Bax, caspase 3, caspase 9, and β-actin by using Moloney murine leukemia virus (M-MuLV) reverse transcriptase and a universal Oligod T primer. The second step involved quantitatively detecting the reverse-transcribed RNA using the GoTaq® 1-stage RT-qPCR System kit, which required particular primers for RNAs of ovarian tissues of the rats (Table 1).

**Table 1:** Primer sets of the studied genes in the current study

Gene	Sense	Antisense	Accession number
*Bax*	5′-AGG ATC GAG CAG AGA GGA TGG- 3′	5′-GAC ACT CGC TCA GCT TCT TGG- 3′	NM_017059.2
*Bcl- 2*	5′-TGT GGA TGA CTG AGT ACC TGA ACC- 3′	5′-CAG CCA GGA GAA ATC AAA CAG AGG- 3′	*NM_016993.1*
*Caspase 3*	5′-ATC CAT GGA AGC AAG TCG AT- 3′	5′-CCT TTT GCT GTG ATC TTC CT- 3′	NM_012922.2
*Caspase 9*	5′-GTG AAG AAC GAC CTG ACT GCT AA- 3′	5′-TCA GCT CAC GTT CAT CGC ATC CG- 3′	NM_031632.1
*β-actin (housekeeping gene)*	5′-TAC AGC TTC ACC ACC ACA GC- 3′	5′-GGA ACC GCT CAT TGC CGA TA- 3′	NM_031144
*miRNA*			
*miRNA 204 - 5p*	5′-ACA CTC CAG CTG GGT TCC CTT TGT CAT CCT AT- 3′	5′-CTC AAC TGG TGT CGT GGA- 3′	MIMAT0000237
*U6 (housekeeping gene)*	5′-CTC GCT TCG GCA GCA CA- 3′	5′-AAC GCT TCA CGA ATT TGC GT- 3′	NM_012321

#### miRNA204 - 5p

The purification of miRNA from ovarian tissues of the rats was accomplished using the mirVana TM miRNA Isolation Kit (Catalogue number: AM1561, Thermo Fisher Scientific, MA, USA) since traditional RNA isolation techniques are not well suited for the isolation of small RNAs. To purify either total RNA or RNA enriched for small species from cell or tissue samples, the kit uses an organic extraction process followed by immobilization of RNA on glass-fiber filters. The benefits of both organic and solid-phase extraction are included in the mirVana miRNA isolation process, while the drawbacks of each are left out. The amount and quality of rats’ ovarian miRNA were performed using a T80 UV/VIS spectrometer as previously described. In addition, All-in-One™ miRNAqRT-PCR Detection Kit 2.0 (Catalog number. QP115, Gene Copoeia Inc. Rockville, MD USA) was used for quantitative detection of mature miRNA. The All-in-One™ miRNA qRT-PCR Detection Kit 2.0 uses real-time PCR technology to quantitatively measure miRNAs. The quantitative determination of rats’ ovarian miRNA- 204- 5p includes two major steps: the first step is the synthesis of cDNA complementary to rats’ ovarian miRNA- 204 - 5p which is done by the use of poly A polymerase to add poly-A tails to the 3′ end of miRNAs, followed by using Moloney murine leukemia virus (M-MuLV) reverse transcriptase and a unique Oligod T adaptor primer to reverse transcribe the miRNA tailed poly-A. The second step is the quantitative detection of the reverse-transcribed miRNA by using the All-in-One TM qPCR Mix containing SYBR® Green which needs specific primer for rats’ ovarian miRNA- 204- 5p (Table 1). The used standard protocol for qPCR was a single step for initial denaturation (at 95 °C for 10 min) and 40 cycles consisting of heat denaturation (at 95 °C for 10 s), primer annealing (at 55 °C for 20 s), and DNA synthesis (at 72 °C for 20 s).

### Computed Calculation of the Relative Expression Levels of mRNAs and miRNA- 204 - 5p in the Ovarian Tissues of Rats

The quantification of mRNA relative expression levels of Bax, Bcl- 2, caspase 9, caspase 3, and miRNA- 204 - 5p was computed using the Applied Biosystem Software, which is integrated into the Applied Biosystems Real-Time PCR Instruments (Thermo Fisher Scientific, Waltham, MA USA). The Ct values of the ovarian mRNAs and miRNA- 204–5p genes in rats that were examined in relation to the housekeeping genes β-actin and U6 respectively are included in the PCR data sheet. It is best to assess the level of gene expression using a control sample. The delta-delta Ct (ΔΔCt) computation is used to quantify and normalize the relative expression of the target gene to its corresponding housekeeping gene. We determined the relative expression of the target gene using 2 ΔΔCt.

### Histopathological Examination

The histological investigation in the current study was undertaken, as started earlier. The ovarian and uterine tissue samples were first fixed using neutral buffered formalin 10%. After that, the samples were dehydrated using ethanol at varying percentages, embedded in paraffin and sectioned at a thickness of 2 to 5 µm. Hematoxylin–eosin (H&E) was then used to stain the samples. Ultimately, a LEICA light microscope equipped with 10 and 20 objective lenses was used to examine these stained films for histological discoveries and demonstrate the morphometric analysis [58].

Furthermore, histological lesions were assessed by examining five randomly fields/sections/organ. The observations were documented in a table that provided an explicit description of the kind, frequently, and intensity of the lesions in each group [59].

### Statistical Analysis

IBM SPSS Statistics 20 was used to apply all the data for statistical analyses in this study. The normal distribution of data was validated by the Shapiro–Wilk test, then a one-way ANOVA test was employed, and for multiple comparisons, the Tukey post hoc test was utilized next. The means and standard error of the means (SEM) of each value were reported. Concerning data of estrus regularity, Fisher’s exact test was applied. The output data’s significant differences were taken into account when the *p* value was less than 0.05.

## Results

### Phytochemical Inspection and In Vitro Antioxidant Potential of *Syzygium cumini* Fruit Extract

As shown in Table 2, findings of in vitro antioxidant power and phytochemical assessment revealed that the total amounts of flavonoid of the SCFE were either 12.94 ± 2.8 or 21.25 ± 5.2 mg/g dried fruit extract, represented as QU and gallic acid (GA) equivalents, respectively. Table 2 further indicated that DPPH scavenging potential of SCFE exhibited notable antioxidant capacity as the effective concentration 50 (EC_50_) value was found to be 49.75 µg/mL. Additionally, the assay of phosphomolybdate complex displayed strong antioxidant power of SCFE (19.35 ± 0.40 mg ascorbic acid equivalent/g dry SCFE).

**Table 2:** Phytochemical analysis and assay of in vitro antioxidant activities of the *Syzygium cumini* fruit extract

Item	Result (mean ± SEM)
I. Phytochemical analysis	
1. Total phenolic content	21.25 ± 5.2 mg/g dry extract expressed as gallic acid equivalent
2. Total flavonoids content	12.94 ± 2.8 mg QU/g dry extract
II. Antioxidant activities assays	
a. Using DPPH scavenging activity assay method	EC50 value was 49.75 µg/mL
b. Using phosphomolybedate complex assay	19.35 ± 0.40 mg ascorbic acid equivalent/g dry extract on the phosphomolybdate complex assay

#### The Metabolites Approaches Based on LC–MS of *Syzygium cumini *Fruit Extract

**Fig. 1:**
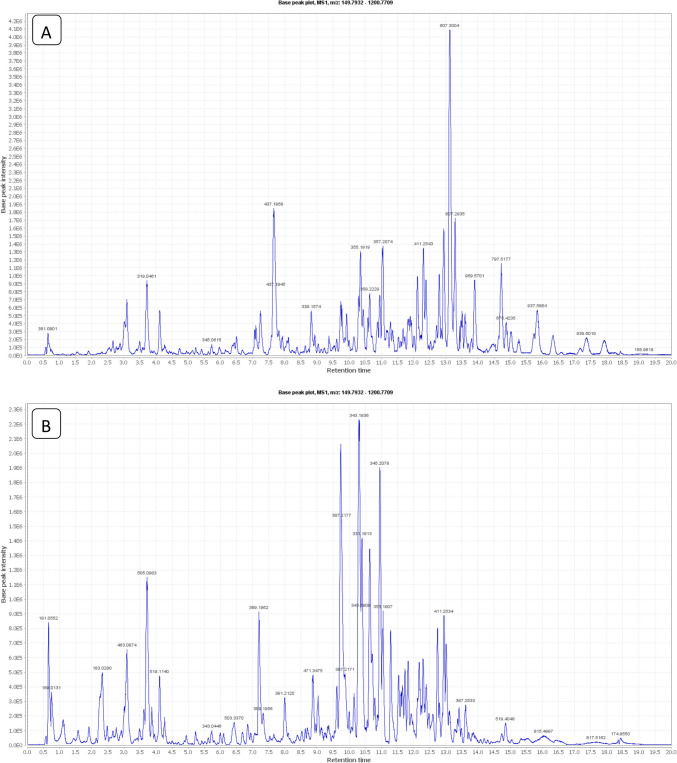
LC/MS total ion chromatogram (**A** positive mode, **B** negative mode) of the SCFE

Using LC-HRMS, metabolite profiling of SCFE was carried out. By utilizing high-resolution MS, RT, and UV data, the databases could help for tentative identification of the dereplicated compounds. Fifteen identified compounds have a chemo-diversity, mainly, anthocyanins, flavonoids, phenolic acids, and their derivatives, which were dereplicated in agreement with the previously isolated and identified compounds from the investigated fruit (Fig. 1; Table 3).

**Table 3:** Annotated compounds by LC–MS metabolite analysis of the *Syzygium cumini* fruit extract

	Ionization	RT	Experimentally Accurate m/z	Theoretically mass m/z	Tentative identification	Ref
1	P	1.040	170.0215	170.0208	Gallic acid	[110]
2	P	2.319	184.0372	184.0373	Methyl gallate	[110]
3	P	4.539	286.0477	286.0483	Kaempferol	[110]
4	P	4.539	287.0556	287.0550	Cyanidin	[111, 112]
5	P	2.310	302.0066	302.0063	Ellagic acid	[110]
6	P	3.876	302.0427	302.0430	quercetin	[110]
7	P	3.824	318.0376	318.0380	Myricetin	[110]
8	N	3.145	465.1029	465.1028	Delphinidin 3-glucoside	[111, 112]
9	P	3.741	506.1052	506.1060	Myricitrin 3-O-(4-O-acetyl)-rhamnoside	[110]
10	N	3.800	507.1136	507.1133	Delphinidin 3-(acetyl-glucoside)	[111, 112]
11	N	4.047	520.1210	520.1217	3,3',5,5',7-Pentahydroxy- 4'-methoxy 3-O-(4-O-acetyl- rhamnopyranosyl- flavone	[110]
12	N	2.801	540.1843	540.1838		[113]
13	N	2.925	626.1483	626.1483		[110]
14	N	4.353	658.1169	658.1170		[110]
15	N	1.973	784.0759	784.0763		[113]

#### Body Weights, Ovarian Weights, and Gonadosomatic Index

**Fig. 2:**
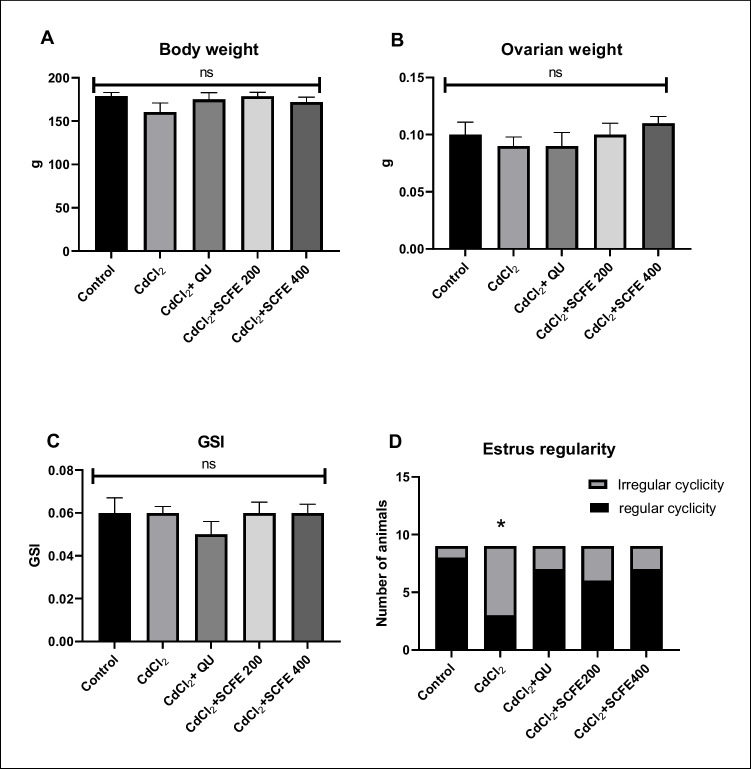
Effects of *Syzygium cumini* fruit extract and quercetin administration on the body weight (**A**), ovarian weight (**B**), gonadosomatic index (**C**), and estrus regularity (**D**) in control and treated rats. g, gram; GSI, gonadosomatic index; QU, quercetin; SCFE, *Syzygium cumini* fruit extract. Values are represented as mean ± SEM (standard error of the mean). ns, means no significant difference (*P* > 0.05) between groups. In **D**, data are presented as a number of rats with regular and irregular estrus cycles (out of 9 female rats). Fisher’s exact test was applied at *P* < 0.05. *Different from the control group (*P* < 0.05)

Rats treated with CdCl_2_ showed comparable body and ovarian weights to the other groups (*p* > 0.05), as Fig. 2 shows. Furthermore, all groups showed consistency in the GSI (*p* > 0.05).

#### Regularity of Estrus Cycle

Figure 2 shows that CdCl_2_ significantly reduced the number of animals with regular estrus cycles (3/8) in relation to the control group (8/8) (*p* < 0.05). Even though QU (7/8), SCFE200 (6/8), and SCFE400 (7/8) improved estrus cyclicity compared to the CdCl_2_ group, the difference was not statistically significant (*p* > 0.05).

#### Female Sex Hormones

The findings regarding the serum levels of gonadotropins (FSH and LH), AMH, and ovarian hormones (estrogen and progesterone) are demonstrated in Table 4. CdCl_2_ treatment significantly lowered the serum levels of FSH, LH, AMH, estrogen, and progesterone compared to the control group (*p* < 0.05).

**Table 4:** Biochemical analyses in different study groups

	Control	CdCl_2_	CdCl_2_ + QU	CdCl_2_ + SCFE200	CdCl_2_ + SCFE400
FSH (ng/mL)	9.30 ± 0.383	3.70 ± 0.25*	5.03 ± 0.083*,**	5.93 ± 0.071*,**	6.92 ± 0.102*,**,***,#
LH (ng/mL)	3.2 ± 0.13	0.96 ± 0.062*	1.73 ± 0.029*,**	2.05 ± 0.025*,**,***	2.38 ± 0.036*,**,***,#
AMH (ng/Ml)	1.9 ± 0.077	0.27 ± 0.019*	1.12 ± 0.019*,**	1.32 ± 0.016*,**,***	1.54 ± 0.023*,**,***,#
Estrogen (ng/L)	15.5 ± 0.196	4.77 ± 0.223*	8.5 ± 0.211*,**	11.11 ± 0.248*,**,***	13.21 ± 0.170*,**,***#
Progesterone (ng/mL)	2.23 ± 0.182	0.75 ± 0.071*	1.31 ± 0.101*,**	1.72 ± 0.029*,**	1.88 ± 0.054**,***
TAC (mmol/g tissues)	1.91 ± 0.04	0.86 ± 0.03*	1.17 ± 0.07*,**	1.22 ± 0.05*,**	1.45 ± 0.05*,**,***,#
SOD (U/g tissues)	2.09 ± 0.06	1.00 ± 0.05*	1.33 ± 0.07*,**	1.41 ± 0.056*,**	1.67 ± 0.08*,**,***
CAT (U/g tissues)	2.52 ± 0.06	1.23 ± 0.07*	2.00 ± 0.10*,**	1.68 ± 0.06*,**	1.59 ± 0.09*,**,***
GSH (mg/g tissues)	2.97 ± 0.09	1.47 ± 0.07*	1.75 ± 0.09*	1.84 ± 0.06*,**	2.16 ± 0.09*,**,#
MDA (nmol/g tissues)	0.79 ± 0.05	2.72 ± 0.10*	1.84 ± 0.05*,**	1.75 ± 0.04*,**	1.45 ± 0.09*,**

Values are represented as mean ± SEM (standard error of the mean)

*FSH* follicle-stimulating hormone, *LH* luteinizing hormone, *AMH* anti-mullerian hormone, *TAC* total antioxidant capacity, *SOD* superoxide dismutase, *CAT* catalase, *GSH* reduced glutathione, *MDA* malondialdehyde

^*^p < 0.05 versus the control group, **p < 0.05 versus CdCl_2_ group, ***p < 0.05 versus CdCl_2_ + QU group, #*p* < 0.05 versus CdCl_2_ + SCFE 200 group. Using one-way ANOVA followed by Tukey’s post hoc test

Interestingly, when rats were co-administered SCFE200, SCFE400, or QU with CdCl_2_, the serum levels of all hormones were significantly (*p* < 0.05) improved in comparison to the CdCl_2_ group. Both doses of SCFE revealed superiority over the QU (*p* < 0.05) in terms of their effects on LH, AMH, and estrogen. In the case of FSH and progesterone, SCE400 outperformed QU (*p* < 0.05). Furthermore, SCE400 was better than SCE200 in recovering FSH, LH, AMH, and estrogen (*p* < 0.05).

#### Oxidant/Antioxidant Markers in the Ovarian Tissues

The ovarian levels of oxidative indicators are represented in Table 4.CdCl_2_ triggered significant oxidative stress in the ovaries, increasing MDA (3.42-fold, Table S1) and decreasing all study antioxidant indicators [SOD (2.11), CAT (2.04), TAC (2.22), and GSH (2.02)-fold, Table S2] compared to the control group (*p* < 0.05). Conversely, both QU and SCFE, at both doses effectively attenuated these oxidative stress markers, reducing MDA (0.67, 0.64, 0.53 folds, respectively) in ovaries while raising antioxidant levels (*p* < 0.05) in comparison to the CdCl_2_-treated group. However, GSH levels were only enhanced with both doses of SCFE. Additionally, SCFE400 demonstrated superior antioxidant efficacy compared to QU (*p* < 0.05), as evidenced by higher TAC and SOD levels and surpassed SCFE200 (*p* < 0.05), as reflected by greater TAC and GSH.

#### The Ovarian mRNA Relative Expression Levels of Bcl- 2, Bax, Caspases 3, 9, and miRNA- 204 - 5p

**Fig. 3:**
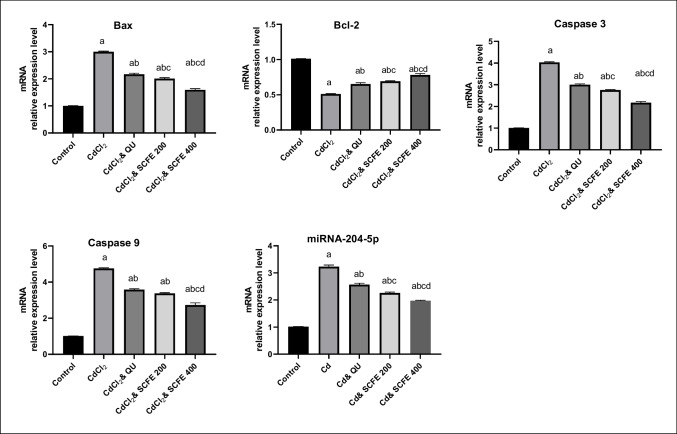
The Ovarian mRNA relative expression levels of Bax, Bcl- 2, miRNA- 204 - 5p, and caspase 3 and caspase 9 in control and treated rats. QU, quercetin; SCFE, *Syzygium cumini* fruit extract. Values are represented as mean ± SEM (standard error of mean). a*p* < 0.05 versus control group. b*p* < 0.05 versus CdCl2 group. c*p* < 0.5 versus CdCl2 + QU group. d*p* < 0.5 versus CdCl2 + SCFE 200 group. Using one-way ANOVA followed by Tukey’s post hoc test

Ovarian Bcl- 2, Bax, caspases 3, 9, and miRNA- 204 - 5pmRNA expression levels are depicted in Fig. 3. Treating female rats with CdCl_2_ resulted in significant upregulation of the mRNA expression levels of Bax (2.98-fold, Table S1), caspases 3 (4.03-fold), 9 (4.70-fold), and miRNA- 204 - 5p (3.21-fold) in ovarian tissues, while Bcl- 2 expression was downregulated (1.99-fold) in comparison to control group (*p* < 0.05).

Most notably, both SCFE and QU dosages were superb candidates to significantly (*p* < 0.05) reduce the adverse effects of CdCl2, resulting in an increase in Bcl- 2 and a decrease in the expression levels of Bax, caspases 3, 9, and miRNA- 204 - 5p.

In terms of reducing the negative effects of CdCl2 on the expression levels of Bax, Bcl- 2, caspase 3, and miRNA- 204 - 5p, both dosages of SCFE outperformed QU (*p* < 0.05). The large dose of the extract (SCFE400) was the treatment that had a greater effect than QU in terms of caspase 9 expression levels (*p* < 0.05). All of the study genes’ expression levels were shown to be improved more by SCFE400 than by SCFE200 (*p* < 0.05).

##### Histopathological Findings

**Fig. 4:**
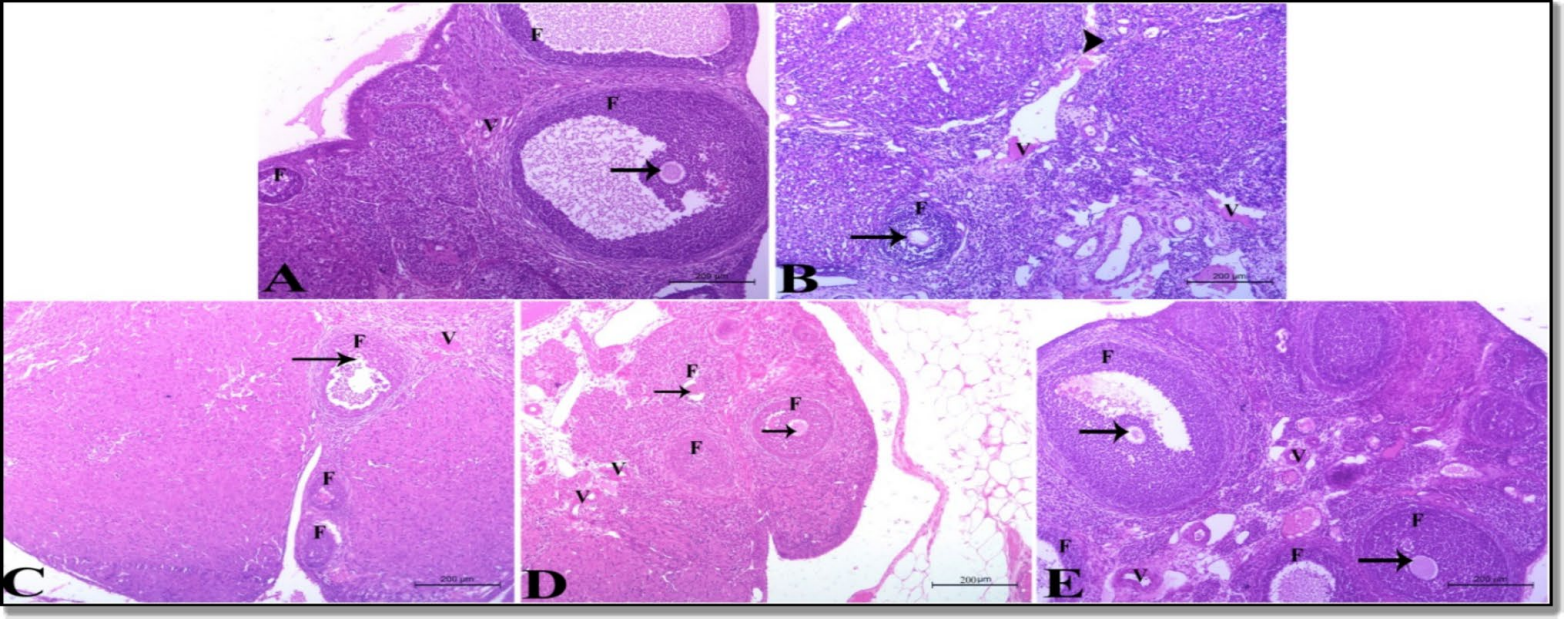
A photomicrograph of ovary of adult female albino rats showing **A** a photomicrograph of control group showed normal ovarian tissue with mature and growing follicles (F) and contained normal oocyte (arrow) as well as normal ovarian blood vessels (V). (H&E) stain × 100. **B** A photomicrograph of CdCl_2_-treated group exhibited growing ovarian follicles (F) that appeared in pre mature state containing degenerated oocytes (arrow). The ovarian blood vessels (V) appeared highly congested. (H&E) stain × 100. **C** A photomicrograph of CdCl_2_ + quercetin-treated group showed growing follicles (F) containing degenerated oocyte (arrow) and congested blood vessels (V) (H&E). Stain × 100. **D** A photomicrograph of CdCl2 + low dose group of *Syzygium cumini* fruit extract (SCFE200) revealed few mature and growing follicles (F) containing normal oocyte (arrow) and normal ovarian blood vessels (V). (H&E) stain × 100. **E** A photomicrograph of CdCl2 + high dose group of *syzygium cumini* fruit extract (SCFE400) showed many mature and growing follicles (F) containing normal oocyte (arrow) and normal ovarian blood vessels (V). (H&E) stain × 100

**Fig. 5:**
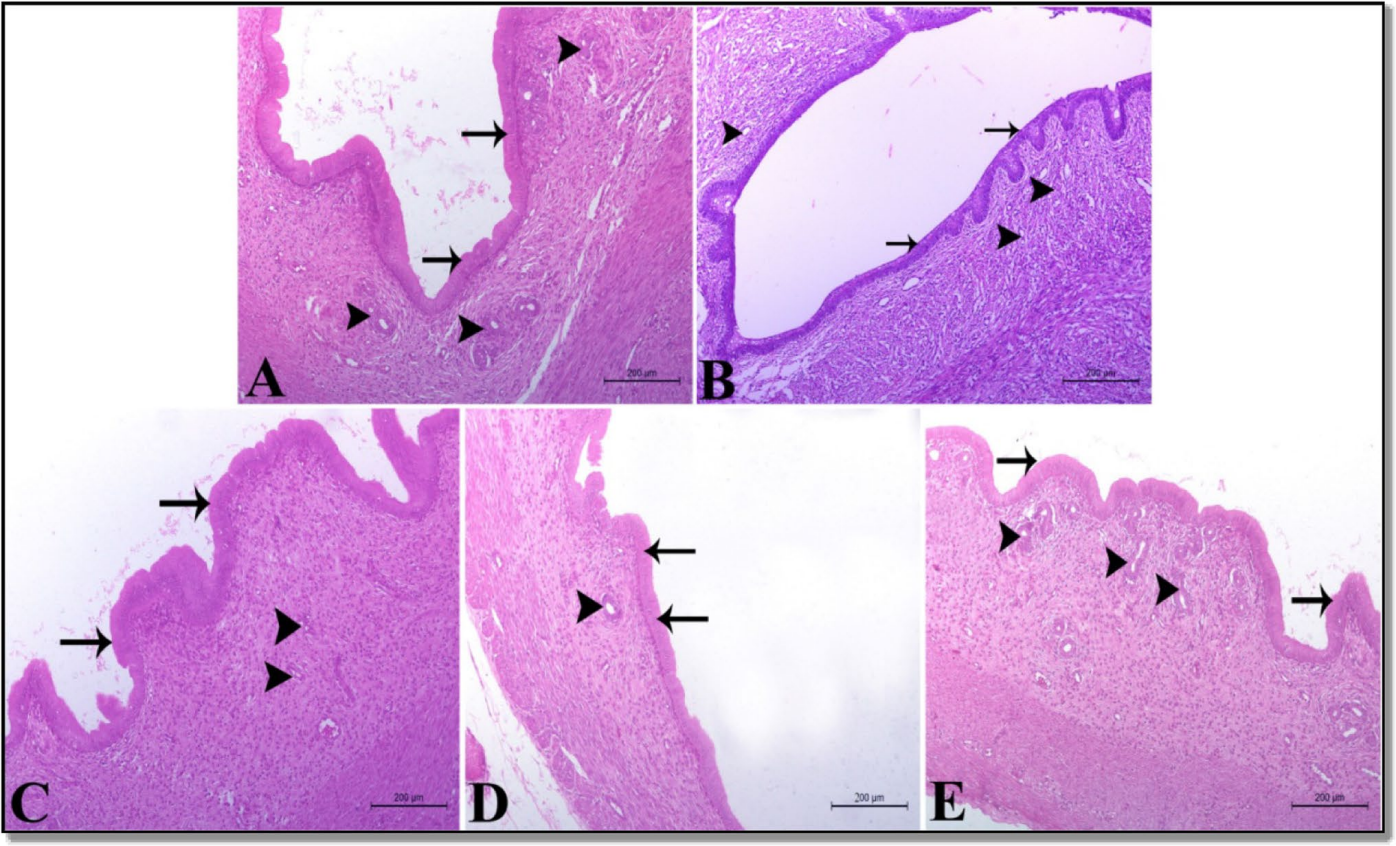
A photomicrograph of uterus of adult female albino rats showing **A** a photomicrograph of control group showed endometrium covered with simple columnar layer (arrow) over thick submucosal layer and contained numerous active endometrial glands (arrowhead). H&E × 100. **B** A photomicrograph of CdCl_2_-treated group showed degeneration and stratification of uterine epithelium (arrow) as well as inactive collapsed endometrial glands (arrow head). (H&E) stain × 100. **C** A photomicrograph of the CdCl_2_ + quercetin-treated group showed normal endometrial epithelium (arrow) and few less active endometrial glands (arrow head). H&E × 100. **D** A photomicrograph of CdCl2 + low-dose group of *Syzygium cumini* fruit extract (SCFE200) showed normal endometrial epithelium (arrow), and the endometrial glands (arrow head) became active. H&E × 100. **E** A photomicrograph of CdCl2 + high-dose group of *Syzygium cumini* fruit extract (SCFE400) showed normal endometrial epithelium (arrow) and the endometrial glands (arrow head) became numerous and highly active. H&E × 100

The histopathological results are illustrated in Figs. 4 and 5. The current study demonstrated that CdCl_2_ induced significant deteriorative changes in ovarian tissues, including the appearance of growing ovarian follicles in a pre-mature state with degenerated oocytes and highly congested ovarian blood vessels (Fig. 4B). Also, it induced degeneration and stratification of uterine epithelium, with the appearance of inactive collapsed endometrial glands (Fig. 5B). Remarkably, all these degenerative changes in ovarian and uterine tissues induced by CdCl_2_ were slightly ameliorated by QU (Figs. 4C and 5C). However, they were greatly ameliorated with SCFE200 as the ovarian tissue showed few mature and growing follicles containing normal oocytes as well as normal ovarian blood vessels (Fig. 4D) while the uterine tissue revealed normal endometrial epithelium and active endometrial glands (Fig. 5D). Furthermore, SCFE400 co-administration produced the best results, with the ovarian tissue displaying many mature and growing follicles containing normal oocytes and ovarian blood vessels (Fig. 4E), as well as the normal endometrial epithelium and endometrial glands becoming numerous and highly active (Fig. 5E).

Moreover, the uterine morphometry data (Table 5) supported the previously reported conclusions of histopathology. In this sense, there was a substantial decrease (*P* < 0.0001) in endometrial thickening, epithelial lining, sub-mucosal thickening, and endometrial gland diameter with CdCl_2_ in comparison to the control uterine picture (*P* < 0.0001). In contrast, QU, SCFE 200, and SCFE 400 treatments significantly enhanced these alterations compared to the CdCl_2_ group.

**Table 5:** Morphometric and histopathological scoring of the ovary and uterus in the treated groups

	Morphometry of the uterine wall				Histopathological scoring		
	Endometrial thickening	Epithelial lining	Submucosal thickening	Diameter of the endometrial glands	Congestion of blood vessels	Activity of the endometrial glands	Follicular atresia
Control	1812.04 ± 16.24b	164.75 ± 1.01b	1569.59 ± 7.72b	114.16 ± 0.33c	0	4	0
CdCl_2_	977.337 ± 11.31 d	150.36 ± 1.01c	719.22 ± 4.15 d	72.25 ± 0.10 d	3	1	3
CdCl_2_ + QU	1273.937 ± 8.99c	153.32 ± 1.12c	853.79 ± 4.66c	116.50 ± 1.01c	1	2	2
CdCl_2_ + SCFE200	1937.07 ± 12.57b	167.14 ± 2.00b	1578.18 ± 11.66b	186.87 ± 1.62b	0	3	1
CdCl2 + SCFE400	2075.77 ± 15.33a	293.51 ± 3.13a	1698.51 ± 10.14a	205.23 ± 1.88a	0	4	0
*p* Value	0.0001	0.0001	0.0001	0.0001			

In the same column, values with different lowercase letters are significant from each other *P* < 0.0001. Histopathological scoring of tissue injury was scaled in degrees as follows: 0 = no change, 1 ≤ 25% tissue damage, 2 = 26–50% tissue damage, 3 = 51–75% tissue damage, 4 = 76–100% tissue damage

Furthermore, scoring of histopathological changes found in the uterus and ovary (Table 5), which highlighted the frequency and severity of lesions seen in various experimental groups was recorded.

## Discussion

The medical community has recognized that infertility is a significant social and medical issue. Heavy metal exposure can impact the reproductive systems of both sexes, increasing the chance of infertility. However, the negative effects of metal exposure and female infertility have rarely been addressed simultaneously [60].

Cadmium can accumulate in humans and animals and impair functions of a range of organs, including liver, kidney, and ovary, with the ovary being the utmost vulnerable organ to its damaging effect [61]. Cadmium adversely affects ovarian tissue by causing oxidative stress [62]. Oxidative stress is represented by stress markers such as MDA [63], which is considered the principal marker for oxidative stress in ovarian tissue [64].

In this investigation, CdCl_2_ administration resulted in a significant rise in MDA, as well as a reduction in all examined antioxidant markers (*p* < 0.05). The results of Abdel-Wahab et al. [53] confirm our findings. Furthermore, rats treated with CdCl2 demonstrated an enhanced formation of free radicals, resulting in a substantial decrease in TAC and a significant increase of MDA in the ovarian tissues [62]. Many investigations either in vitro or in vivo have proven that Cd interferes with antioxidant defense by reducing antioxidant levels, which are crucial for ROS elimination [65].

It is interesting to note that the oxidative stress triggered by Cd in the ovarian tissues was markedly (*p* < 0.05) reversed by coadministration of either QU or both doses of SCFE with CdCl_2_. The fold change data (Table S2) revealed that the ameliorating effect of QU, SCFE200, and SCFE400 was 1.33-, 1.42-, and 1.68-fold, respectively, for SOD; 1.28-, 1.36-, and 1.62-fold, respectively for CAT; 1.19-, 1.25-, and 1.47-fold, respectively, for GSH; and 1.37-, 1.42-, and 1.69-fold, respectively, for TAC, reflecting the superior activity of the high dose of the extract.

These findings are consistent with earlier research that demonstrated QU’s protective effect against Cd toxicity and restored the typical oxidative state of ovarian tissues [66]. In an in vitro experiment, QU increased the expression of genes related to CAT and SOD activities [67]. Scavenging peroxides like H_2_O_2_ is a critical function of CAT and SOD, which are capable of catalyzing the dispersion of superoxide anion radicals to create oxygen and hydrogen peroxide [68]. Another study conducted by Nna et al. [54] confirmed that the concurrent administration of QU and CdCl_2_ demonstrated protection against oxidative stress. That oxidative stress was evidenced by the noteworthy elevation of antioxidants and the remarkable reduction of H_2_O_2_ and MDA concentrations in the ovaries when compared to the CdCl_2_ group.

We hypothesize that the active ingredients of SCFE with their strong antioxidant properties are primarily responsible for the ameliorative effects of the extract against Cd-induced ovarian oxidative stress. Our phytochemical investigation revealed that every gram of dried SCFE contained 12.94 ± 2.8 mg of QU equivalents, and our LC–MS assessment revealed the existence of 15 active ingredients including QU. According to a study of Ding et al. [66], QU, an active ingredient of SCFE, effectively reduced the oxidative stress that Cd caused. In addition, Kaempferol (another active ingredient of SCFE) reduces oxidative stress, inflammation, and apoptosis in the hippocampus tissues triggered by CdCl2 [69]. Moreover, supplementing mice’s diet with cyanidin- 3-O-glucoside, an additional SCFE component, enhanced SOD and GSH activities and reduced lipid peroxidation activities that were impeded by CdCl2 [70]. Because cyanidin inhibits the activity of the enzyme xanthine oxidase, which produces ROS, it was thought to be a promising antioxidant [71]. Furthermore, in rats with polycystic ovarian syndrome (PCO), gallic acid “GA” (another ingredient of SCFE) was observed to be a strong antioxidant by lowering the quantity of oxidative challenges in ovarian tissue [72]. Therefore, as demonstrated by the results of the current LC–MS analysis, the protective benefits of SCFE against Cd-induced oxidative insult were mostly due to the antioxidant activity of its constituents, namely, QU, cyanidin, Kaempferol, and GA.

The current study showed that CdCl_2_ had no significant effects on body and ovarian weights, as well as GSI, as compared to other groups. This is in line with earlier findings of Abdel-Wahab et al. [53]. In addition, it has been reported that there were no statistical significances in the BWG and ultimate body and ovarian weights in Cd-treated rats related to control ones [73]. However, these findings differ from those reported by Farombi et al. [74], revealing a decrease in body weight following oral Cd administration by a dose of 15 mg/kg. Also, Cd used by doses of 50 and 200 mg/L resulted in a reduction in both body and ovarian weights [75]. Since the doses utilized in these investigations were higher than the dosages used in our investigation (5 mg/kg BW), we may infer that the effects of Cd on ovarian and body weights are dose-dependent.

The data in the current study demonstrated that CdCl_2_ significantly decreased the levels of gonadotropins (FSH, LH) compared to the control group. Calculation of fold changes (Table S1) revealed that CdCl_2_ recued FSH, LH, AMH, estrogen, and progesterone levels by 2.52-, 3.34-, 6.94-, 3.25-, and 2.9-fold, respectively, in comparison to the control group. This is in line with the findings of a previous study [73], which found that Cd disrupted gonadotropin hormone levels, which were associated with disruption of ovarian follicles. Numerous studies have demonstrated that Cd may disrupt pituitary and endocrine processes [76–78]. The pituitary glands of rats may easily absorb and store Cd resulting in a change in the hypothalamic-pituitary axis (HPA) typical function [76]. Because of this, there was a decrease in the synthesis of GnRH, which acts on receptors in the anterior pituitary gland of the brain to release LH and FSH, causing a drop in FSH and LH levels [73]. Another possible reason for Cd-induced decreases in FSH and LH levels is Cd buildup in the hypothalamus and anterior pituitary, which produced oxidative stress and in turn caused the anterior pituitary’s gonadotrophs to undergo apoptosis.

Since the number of viable gonadotrophs may directly affect the quantities of FSH and LH [54], this could be partially to blame for what resulted in lower blood FSH and LH values in our findings. In addition, reduced levels of FSH and LH were linked to the disturbance of ovarian follicles, as demonstrated by Ruslee et al. [73]. Our histopathological observations further confirmed this, as we observed premature ovarian follicles with degenerated oocytes and highly congested ovarian blood vessels.

Since the anterior pituitary gland secretes FSH and LH, which stimulate the ovaries to secrete estrogen and progesterone respectively, these hormones become necessary for the biosynthesis of estrogen and progesterone [79]. Our findings showed that in comparison to the control group, CdCl_2_ had significantly lower levels of estrogen and progesterone. This aligns with the findings of previous reports [76, 80]. Steroid hormones like estrogen and progesterone play a crucial function in the differentiation and development of reproductive tissues as well as the maintenance of fertility [73]. Since less FSH in the blood may prevent immature oocytes from maturation, this may be partially accountable for the observed drop in estrogen concentrations in the CdCl_2_ group. Furthermore, suppressing the maturation process results in the generation of minute numbers of corpora lutea. Thus, insufficient corpus luteum inhibits progesterone from increasing growth [79].

This study also showed that therapy with CdCl_2_ had an adverse impact on AMH by decreasing its levels in the bloodstream, which aligns with the findings of Lee et al. [81]. In this context, it has been noted that Cd exposure to female rats resulted in polycystic ovarian syndrome and premature ovarian failure through decreasing AMH levels [76]. Increased levels of heavy metals such as mercury and Cd were also linked to faster rates of AMH depletion over time [82]. Since one of the primary targets of Cd in the ovaries is granulosa cells which are the primary somatic cells that make up ovarian follicles [83] and are responsible for the production of AMH [83], Cd’s toxic effects on such cells were shown to be potent in lowering serum AMH levels, which validate our findings. This would lead us to hypothesize that exposure to Cd may impair ovarian reserve via reducing serum levels of AMH, which in turn lowers female reproductive capacity.

According to our findings, SCFE and QU protect against the detrimental effects of Cd on all studied hormones. Calculated fold changes (Table S2) revealed that QU improved the levels of FSH, LH, AMH, estrogen, and progesterone by 1.36-, 1.81-, 4.07-, 1.78-, and 1.75-fold, respectively. Moreover, they were 1.60-, 2.14-, 4.80-, 2.33-, and 2.30-fold, respectively, for SCFE200 and 1.87-, 2.49-, 5.61-, 2.77-, 2.51-fold, respectively, for SCFE400 compared to CdCl_2_. This confirmed the superior improving efficacy for SCFE400 on such hormones. A study by Nna et al. [54] confirmed the beneficial impacts of QU on female reproductive hormones (estrogen, progesterone, FSH, and LH) against Cd toxicity by returning their levels to normal. This could be attributed to the ability of QU to greatly reduce oxidative stress in ovarian tissues. In that study, the remarkable improvement in the hormonal profile is primarily caused by QU’s capacity to decrease Cd buildup by either changing the gut’s absorption of CdCl_2_ or improving the kidneys’ ability to excrete it and the resulting rise in antioxidants in the ovaries. This appears to be correct since the QU-treated groups showed a large decrease in apoptosis, one of the classic indicators of oxidative stress. This means that more live granulosa cells were able to respond to FSH and LH stimulation, which in turn increased steroidogenesis. Moreover, previous studies have connected QU’s metal chelating capacity to its effectiveness in reducing oxidative stress caused by non-essential metals [77]. Regarding the positive impacts of SCFE, it has been reported that serum levels of LH, FSH, and estrogen were considerably lower in rats treated with Cd, but they greatly increased after receiving GA therapy [77]. Previous research revealed that the compounds QU and kaempferol which are major constituents in SCFE had the ability to scavenge free radicals, and the estrogenic properties of QU and kaempferol would compete with Cd’s metalloestrogens for binding to estrogen receptors [84–86]. Thus, the active constituents of SCFE, such as GA, QU, and kaempferol, provide an explanation for why the female hormones maintained normal with SCFE against Cd poisoning.

Our finding illustrated decreased estrus regularity with CdCl_2_. In this context, elevated levels of Cd led to structural damage in the ovaries, disruptions in the estrous cycle, endometrial shrinkage, and a marked drop in estrogen content in the uterus and plasma as well as lipid peroxidation [87]. Additionally, our findings revealed beneficial roles for QU and SCFE in amending estrus cycle regularity; however, it was not significant. This could be attributed to the antioxidant properties of these treatments and their positive impacts on the hormonal state. According to Jose et al. [88], QU was noticed to have a Cd-relieving effect on the regularity of the estrus cycle, which corroborates our findings.

When oxidative stress causes cell death, apoptosis is thought to be a key factor. Numerous investigations have shown that Cd causes apoptosis both in vivo and in vitro [89, 90]. We found in our study a rise in the mRNA relative expression level of the proapoptotic Bax and a decrease in the mRNA relative expression of the antiapoptotic Bcl- 2 after Cd administration. In agreement with our findings, a rise in the Bax to Bcl- 2 ratio with Cd, indicating follicular cell death, which could be attributed to oxidative stress in the ovaries and hormonal dysregulation, was previously reported [54].

To the best our knowledge, this study is the first to investigate the effect of quercetin and *Syzygium cumini* extract on the cadmium-induced altered relative expression of one of the ovarian microRNAs, which is miRNA- 204 - 5p. It also elucidated their effects on the molecular pathway triggered by this miRNA, especially those of apoptosis. Herein, miRNA- 204 - 5p expression was increased with Cd (3.21-fold, Table S1) compared to the control group. miRNA- 204 - 5p is one of the miRNAs involved in intrinsic apoptosis and produces an imbalance in the amounts of apoptotic and antiapoptotic proteins. Cadmium has the ability to cause apoptosis via inhibiting the Bcl- 2 gene expression, which is regulated by the miRNA (MiR- 204 - 5p). When it binds to Bcl- 2, it can induce apoptosis in rat ovarian granulosa cells; nevertheless, its suppression can guard against the ovarian toxicity of Cd. To reduce Bcl- 2 gene expression at the post-transcriptional level, miRNA- 204 - 5p targets crucial elements in the apoptotic signaling cascade [21, 91].

The highly coordinated and dynamic process of megapore formation is caused by intrinsic apoptotic stimuli or by proteins like Bak (Bcl- 2 antagonist/killer) and Bax (Bcl- 2 associated X protein), which are proapoptotic proteins. They interact with tBid (activated bid), which activates Bak and Bax and causes pores in the outer mitochondrial membrane [92].

Furthermore, the research of Su et al. [93] verified the impact of miR- 204 - 5p on the Bax protein by elevating its expression. This causes the release of cytochrome C from mitochondria, which in turn triggers the activation of caspase- 9 and caspase- 3 to start the process of cell death [94].

It is noteworthy that CdCl_2_’s apoptotic effects could be mitigated by QU and SCFE. The estimated fold changes (Table S2) revealed that the decrement effects of QU, SCFE200, and SCFE400 were 0.73-, 0.67-, and 0.53-fold, respectively, for Bax; 0.74-, 0.68-, 0.54-fold, respectively, for caspase 3; 0.75-, 0.71-, and 0.57-fold, respectively, for caspase 9; 0.79-, 0.70-, and 0.61-fold, respectively, for miRNA- 204 - 5p. The enhancing effects of the treatments for Bcl- 2 were 1.28-, 1.36-, and 1.53-fold, respectively, in relation to the CdCl_2_ group, reflecting the outperformancy of the high dose of the extract.

In line with our findings, it has been reported that QU prevents the activation of the apoptotic pathway through a process mediated by free radicals and by decreasing Bax/Bcl- 2 ratio [95]. In addition, QU’s ability to lower miRNA- 204 - 5p expression is confirmed by the findings of Rajabi et al. [96].

Furthermore, the phytochemical composition of SCFE is primarily responsible for its antiapoptotic action. Regarding this, kaempferol has the potential to reduce the amount of apoptosis and reverse the levels of Bcl- 2, Bax, and cytochrome c protein expression in the cytoplasm or mitochondria [97]. Additionally, by modifying the MAPK signaling pathway, cyanidin- 3-O-glucoside was able to greatly reduce the apoptotic effect of Cd [98]. Moreover, cyanidin- 3-O-glucoside (C3G) was proved by its efficiency in downregulation of miRNA- 204 - 5p [90].

Caspase- 3 is the executor that triggers apoptosis and is the last common pathway between extrinsic and intrinsic apoptotic processes [54]. In our study, there was a substantial increase in cleaved caspases 3 and 9 activities in the CdCl_2_-treated group. These data agree with previous findings [99]. According to previous studies [100, 101], Cd-induced apoptosis was facilitated by caspase- 9 activation, a crucial marker of apoptosis brought on via the mitochondrial route. Caspase- 9 activation occurs by apoptosome which is a complex of cytochrome c, Apaf- 1, and procaspase 9, then caspase 9 cleaves procaspase 3 into caspase 3 resulting in apoptosis. Supporting our data, the mRNA relative expressions of caspase- 9 and caspase- 3 in renal tissue were dramatically enhanced by Cd exposure, whereas the mRNA relative expressions of the same enzymes were significantly lowered in the QU-treated group [99]. Concerning SCFE, these findings may be connected to the active components in SCFE. In this regard, the extract’s QU content has the ability to decrease caspase levels that are produced through Bax induction by Cd [100]. Myricetin, an active component of SCFE, demonstrated antiapoptotic activity in H_2_O_2_-treated cells by raising antiapoptotic factor Bcl- 2 and lowering proapoptotic agents Bax and caspases 9 and 3 [102]. Another active ingredient, cyanidin, was observed to ameliorate the apoptosis triggered by cisplatin via the decrement of caspases 3 and 9 expression along with an increment of Bcl- 2, which validate our results [103]. According to a previous study [104], two additional anthocyanin components found in SCFE, cyanidin- 3-Oglucoside and delphinidin- 3-Oglucoside, are more effective antiapoptotic agents. They exerted antiapoptotic effect by binding to the BIR2 region and allosteric sites in caspase 3 and inhibited them through hydrophobic and hydrogen bond interactions. Cyanidin enhanced the ratio of Bcl- 2/Bax in a concentration-dependent manner while decreasing the expression of Bax, cytochrome c, cleavage caspase- 9, and cleavage caspase- 3 [105].

Because no prior research has demonstrated the precise mechanism of SCFE’s antiapoptotic effects, our study is considered the first to confirm the plant’s beneficial antiapoptotic effects by downregulating miRNA- 204 - 5p, modulating Bax/Bcl- 2 expression, and lowering caspase level (caspases 3 and 9). In summary, the 15 bioactive ingredients of SCFE including QU, Kaempferol, cyanidin- 3-O-glucoside, gallic acid “GA,” anthocyanins, flavonoids, phenolic acids, and their derivatives synergistically act as potent antioxidants [106]. They performed their action by decreasing the generation of reactive oxygen species such as H_2_O_2_ that induced oxidative stress which was manifested by increment of lipid peroxidation indicator, MDA, and decrement of antioxidants including GSH, TAC, SOD, and CAT. This oxidative damage induced ovarian apoptosis [106]. Conclusively, these ingredients constituted umbrella of strong antioxidants that succeeded in alleviating cadmium-induced oxidative stress and apoptosis in the ovarian tissues.

Furthermore, previous reports validate beneficial effect of QU and SCFE on human health. In this context, oral QU supplements improved metabolic profiles in patients with polycystic ovarian syndrome [107]. Quercetin has an important role in suppression of ovarian cancer growth and induction of apoptosis [108]. The anticancer efficacy of phytochemicals obtained from *S. cumini* in ovarian cancer cells was validated by investigation [109]. This prompts us to further explore the potential advantageous effects of both SCFE and QU on human health.

Our histopathological examinations as well as the morphometric and histopathological scoring of the ovary and uterus highlighted the harmful effect of Cd on ovarian and uterine tissues, and this is confirmed by the findings of Nasiadek et al. [87]. In addition, the histopathological findings proved the protective impacts of either QU or SCFE on female fertility. A previous study confirmed the protective roles of QU on the ovarian and uterine histological picture [54]. The antioxidant capabilities of QU and SCFE were principally responsible for their positive effects against Cd-induced alteration in the histology of ovarian and uterine tissues.

The limitations of the present study were the animal models, the issue of shortage of evaluated dosages, and the shortage of financial budget to cover extra measurements. Despite these limitations, this in vivo study illustrated for the first time the role played by miRNA- 204 - 5p in cadmium-induced ovarian apoptosis. In that the cadmium upregulated miRNA- 204 - 5p that in turn encouraged ovarian apoptosis through downregulation of antiapoptotic gene; Bcl- 2 and upregulation of apoptotic genes; Bax and caspases 3 and 9. Fortunately, the QU and SCFE (in its two dosages) succeeded in reducing ovarian apoptosis through amelioration of alterations of the expression of miRNA- 204 - 5p, Bcl- 2, Bax, and caspases 3 and 9 (Fig. S2).

## Conclusion and Future Perspectives

In conclusion, the study found that SCFE, a rich source of flavonoids and antioxidants as evidenced by LC–MS analysis, has potential as a therapeutic agent for mitigating CdCl_2_-induced reproductive toxicity that was manifested by irregular estrus cycles, hormonal imbalance, oxidative stress, and ovarian apoptosis. The extract showed significant efficacy in restoring reproductive function, improving estrus cyclicity, hormonal balance, and downregulating miRNA- 204 - 5p expression that encouraged upregulation of an antiapoptotic marker (Bcl- 2) and downregulation of apoptotic markers (Bax and caspases 3 and 9) and improvement of ovarian and uterine histology. SCFE and QU effectively mitigated the adverse effects of CdCl_2_, with SCFE 400 showing superior efficacy. Further research is planned to isolate and characterize specific bioactive compounds responsible for these effects and conduct clinical trials to evaluate the efficacy and safety of SCFE or its derived compounds in humans exposed to Cd or other environmental pollutants.

## Supplementary Information

Below is the link to the electronic supplementary material.

Supplementary file1 (DOCX 441 KB)

## Data Availability

No datasets were generated or analysed during the current study.
