# Supplementary material for: Syzygium cumini Fruit Extract and Quercetin Ameliorate Cadmium-Induced Ovarian Apoptosis in Rats Via miRNA- 204 - 5p-Mediated Bcl- 2 Upregulation and Bax/Caspase 9/Caspase 3 Downregulation
Source: Biol Trace Elem Res. 2025 May 14;203(12):6172–91. doi: 10.1007/s12011-025-04632-y (PMC12672602; doi:10.1007/s12011-025-04632-y)
Supplement: Supplementary file 1 — Supplementary file1 (DOCX 441 KB) [file 12011_2025_4632_MOESM1_ESM.docx]

**Methods:**

**Phytochemical Analysis and the Assay of In Vitro Antioxidant Activities of Syzygium cumini Fruit Extract (SCFE)**

Phytochemical analysis and an in vitro antioxidant activity experiment were performed on the Syzygium cumini fruit extract at Department of Pharmacognosy, Faculty, of Pharmacy Minia University as follows:

**Phytochemical analysis**

***Determination of total phenols content of SCFE***

Syzygium cumini fruit extract's total phenols content was measured using the procedure outlined by [1]. Consequently, the Folin-Ciocalteu (FC) reagent was employed, and gallic acid was used as a standard to calculate the results.

**A. Steps**

1. 300 μl (20% anhydrous sodium carbonate), 50 μl Folin–Ciocalteu reagent (2N), 3.5 ml deionised water, and 50 μl Syzygium cumini Fruit Extract (10 mg/ml) were combined.

2. After 30 minutes, the absorbance was measured in triplicate at 728 nm in comparison to an absolute methanol blank.

3. Gallic acid was used to create the calibration curve, which allowed the total phenol concentration to be represented in gallic acid equivalent (GAE).

***Determination of total flavonoids content of SCFE***

The total flavonoids content of the Syzygium cumini Fruit Extract was determined using a modified colorimetric method described by [2].

**A. Steps**

1. 300 μl of 5% W/V NaNO_2_ combined with 2 ml of sample (2.5 mg/ml) was used.

2. After six minutes, 300 μl of 10% AlCl_3_ W/V was added, and it was let stand for extra six minutes. One millilitre of 4% W/V NaOH was then added to get a red colour.

3. After mixing the mixture well and letting it rest for 15 minutes, deionised water was added right away to reach the final amount of 6 ml.

4. At 510 nm, the absorption was measured three times. Quercetin was used to create the calibration curve, which allowed the total flavonoid concentration to be represented in quercetin equivalent (QE).

***Antioxidant activities assays of SCFE***

Dry Syzygium cumini fruit extract was redissolved in ethanol (95%) to provide a concentration of 2.5 mg/mL and serial dilution was then made to prepare appropriate concentrations for antioxidant assays.

***diphenyl-2-picryl-hydrazyl (DPPH) radical scavenging activity assay***

The free radical scavenging potentials of the ethanol extract of Syzygium cumini was estimated spectrophotometrically using 1,1-diphenyl-2-picryl-hydrazyl (DPPH) according to the method of [3].

**A. Steps**

1. 200 μl of Syzygium cumini fruit extract (at different concentrations) was mixed thoroughly with 2 ml of DPPH solution (0.1 mM), and the combination was then left in the dark at 25 ˚C for 15 minutes.

2. Regarding the control, ethanol was used instead of the fraction, and the absorbance at 517 nm was measured three times.

3. The graph of the DPPH scavenging action against Syzygium cumini fruit extract concentration was used to determine the concentration that decreased half of the original DPPH concentration (EC50). The standard was vitamin C. (Fig S1).

**Fig. S1.** Graph shows DPPH scavenging effect against *Syzygium cumini* fruit extract concentration

***Phosphomolybdate complex antioxidant capacity assay***

The total reducing abilities of Syzygium cumini fruit extract were assessed by phosphomolybdate complex method, in which the ascorbic acid was used as a standard [3]**.**

**A. Steps**

1. Briefly, 0.3 ml of sample solution (2.5 mg/ml) was mixed with 3 ml of reagent solution (0.6 M sulphuric acid, 28 mM sodium phosphate and 4 mM ammonium molybdate) in capped tubes, which were incubated in a water bath for 90 min at 95 °C.

2. After cooling them, the absorbance was measured at 695 nm against a blank three times.

3. The calibration curve was made using ascorbic acid so that the antioxidant activity was expressed in ascorbic acid equiv. mg/g dry Syzygium cumini fruit extract.

**LC-MS Analysis**

Following a milligram of Syzygium cumini fruit extract being dissolved in methanol to reach a concentration of 1 mg/ml, high-resolution LC-ESI-MS analysis was performed using Thermo Scientific Exact mass analyzers (Thermo Scientific, Karlsruhe, Germany) coupled with a 3000 HPLC system (Dione Ultimate). The samples were eluted using a C-18 column (75 mm × 3.0 mm, 5 μm, ACE, Mainz, Germany). The mobile phase, which was designed to be eluted at a flow rate of 300 µl/min, consisted of acetonitrile and water with 0.1% formic acid. Gradient elution was initiated with 10% acetonitrile for five minutes, then over the course of thirty minutes, it was increased to 100% acetonitrile, which was held for an additional five minutes before falling back to 10% acetonitrile in the sixth minute. 10% acetonitrile was used to reach column equilibration for five minutes, or until the end of the run. Every sample was analyzed for a total of 45 minutes. The tray temperature was kept at 12 ^o^C and the injection volume was 10 µl.

The MS dataset underwent processing and data extraction using MZmine 2.20, according to the predefined settings. Then we performed chromatogram deconvolution followed by peaks deisotoping. Normalization of the retention time was applied for chromatographic alignment and gap-filling [4]. We used the Excel macros to combine positive and negative ionization mode data files generated by MZmine to cover the maximum number of metabolites. The dereplication study against in-house and DNP, METLIN databases was processed using RT and m/z threshold of ±5 ppm, which in turn produced tentative identification of all secondary metabolites in each investigated extract.

**Supplementary results:**


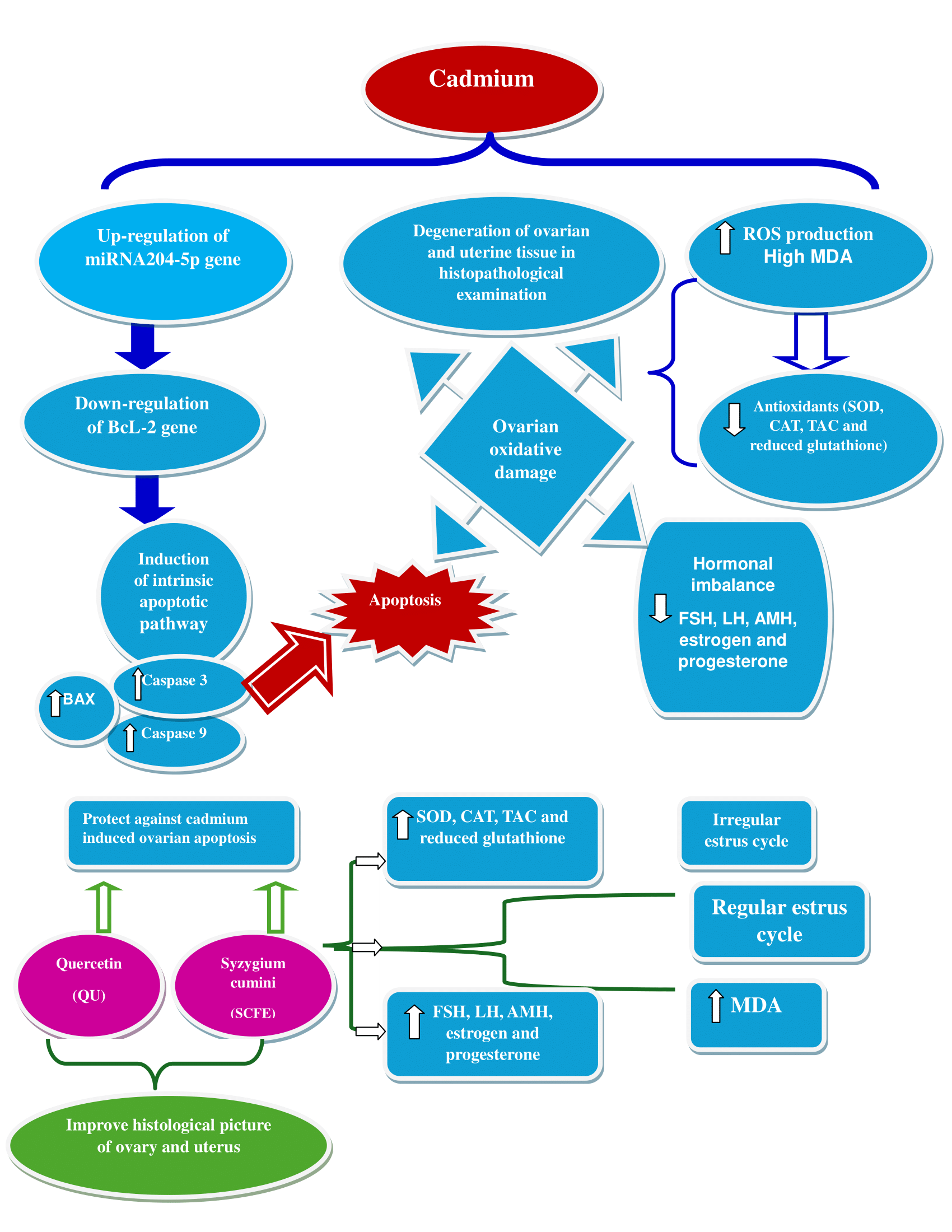


**Figure S2**: A schematic figure showing the molecular mechanism of the protective effect of *Syzygium cumini* fruit extract and qurecetin against cadmium-induced ovarian apoptosis.

| parameter | Control | CdCl_2_ |
| --- | --- | --- |
| FSH (ng/mL) | 1 | ↓2.52 |
| LH (ng/mL) | 1 | ↓3.34 |
| AMH (ng/Ml) | 1 | ↓6.94 |
| Estrogen (ng/L) | 1 | ↓3.25 |
| Progesterone (ng/ml) | 1 | ↓2.98 |
| TAC (mmol/g tissues) | 1 | ↓2.22 |
| SOD (U/g tissues) | 1 | ↓2.11 |
| CAT (U/g tissues) | 1 | ↓2.04 |
| GSH (mg/g tissues) | 1 | ↓2.02 |
| MDA (nmol/g tissues) | 1 | ↑3.42 |
| Bax | 1 | ↑2.98 |
| Bcl-2 | 1 | ↓1.99 |
| Caspase 3 | 1 | ↑4.03 |
| Caspase 9 | 1 | ↑4.70 |
| miRNA-204-5p | 1 | ↑3.21 |

**Table S1: Fold changes of CdCl_2_ in relation to control.**

↓ means that the values are decreased compared to control.

↑means that the values are increased compared to control.

| parameter | CdCl_2_ | CdCl_2_ + QU | CdCl_2_ + SCFE200 | CdCl_2_+SCFE400 |
| --- | --- | --- | --- | --- |
| FSH (ng/mL) | 1 | ↑1.36 | ↑1.60 | ↑1.87 |
| LH (ng/mL) | 1 | ↑1.81 | ↑2.14 | ↑2.49 |
| AMH (ng/Ml) | 1 | ↑4.07 | ↑4.80 | ↑5.61 |
| Estrogen (ng/L) | 1 | ↑1.78 | ↑2.33 | ↑2.77 |
| Progesterone (ng/ml) | 1 | ↑1.75 | ↑2.30 | ↑2.51 |
| TAC (mmol/g tissues) | 1 | ↑1.37 | ↑1.42 | ↑1.69 |
| SOD (U/g tissues) | 1 | ↑1.33 | ↑1.42 | ↑1.68 |
| CAT (U/g tissues) | 1 | ↑1.28 | ↑1.36 | ↑1.62 |
| GSH (mg/g tissues) | 1 | ↑1.19 | ↑1.25 | ↑1.47 |
| MDA (nmol/g tissues) | 1 | ↓0.67 | ↓0.64 | ↓0.53 |
| Bax | 1 | ↓0.73 | ↓0.67 | ↓0.53 |
| Bcl-2 | 1 | ↑1.28 | ↑1.36 | ↑1.53 |
| Caspase 3 | 1 | ↓0.74 | ↓0.68 | ↓0.54 |
| Caspase 9 | 1 | ↓0.75 | ↓0.71 | ↓0.57 |
| miRNA-204-5p | 1 | ↓0.79 | ↓0.70 | ↓0.61 |

**Table S2: Fold changes of the studied treatments in relation to CdCl_2_.**

↓ means that the values are decreased compared to CdCl_2_.

↑means that the values are increased compared to CdCl_2_.

**References:**

1. Vl S (1999) Analysis of total phenols and other oxidation substrates and antioxidants by means of Folin-Ciocalteu reagent. Methods Enzymol 299:152–178

2. Benariba N, Djaziri R, Bellakhdar W, et al (2013) Phytochemical screening and free radical scavenging activity of Citrullus colocynthis seeds extracts. Asian Pac J Trop Biomed 3:35–40

3. Jain A, Roy S, Joshi A, Joshi N (2016) Evaluation of In-vitro cytotoxic and antioxidant activity of methanolic extracts of Ipomoea carnea and Alternanthera sessilis. Int J Bioassays 5:4763–4766

4. Elsayed Y, Refaat J, Abdelmohsen UR, et al (2018) Metabolomic profiling and biological investigation of the marine sponge‐derived bacterium Rhodococcus sp. UA13. Phytochem Anal 29:543–548
